# Suicide and deliberate self-harm among women in Nepal: a scoping review

**DOI:** 10.1186/s12905-021-01547-3

**Published:** 2021-12-09

**Authors:** Sarina Pradhan Kasaju, Anja Krumeich, Marc Van der Putten

**Affiliations:** 1 Faculty of Health, Medicine and Lifesciences, Maastricht University, Universiteitssingel 60, P.O.Box 616, 6200 MD Maastricht, The Netherlands; 2 Faculty of Public Health, Thammasat University, Rangsit Campus, Pathum Thani, 12120 Thailand

**Keywords:** Suicide, Deliberate self-harm, Women, Asia, Nepal, Public health

## Abstract

**Background:**

Suicide is a growing public health problem globally. Suicide accounts for 70% of violent deaths among women in low and middle income countries. In Nepal suicide is the single leading cause of death among women of reproductive age. The aim of this scoping review is to explore and understand the various contexts related to vulnerability of Nepalese woman towards suicide and deliberate self-harm.

**Methods:**

A scoping review based on Arksey and O’Malley’s methodological framework including a combination of peer-reviewed publications and grey literature was conducted. The National Library of Medicine’s PubMed and Google Scholar search engines were used during July 2019 applying a Boolean search strategy.

**Results:**

Suicide incidence was found to be higher among younger age group and married women, with poisoning as the most common means of suicide. Psychosocial and economic factors such as abuse, interpersonal conflicts, marital disputes, relationship problems, adjustment problems, unpaid loans and financial losses; and mental health conditions such as mood disorder, adjustment disorder and substance abuse disorder were found to be contributing factors for suicide and deliberate self-harm among women in Nepal.

**Conclusion:**

Socio-cultural and economic factors shape family and marital relationships which impacts psycho-social and mental wellbeing of women in Nepal inciting suicidal attempts and deliberate self-harm. However, very few studies were found that explore the context of poverty, social exclusion, gender inequality, education, traditional/cultural and patriarchal system in which suicide among women in Nepal occurs.

## Background

Every year an estimated 700,000 people die from suicide globally, roughly corresponding to one death every 40 s [1]. Of all global suicides, 79% occur in low and middle income countries (LMICs) [1]. In LMICs, suicide accounts for 70% of violent deaths among women [2]. As suicide is gradually emerging as a major global public health problem, suicide among men has been given more consideration with relative silence about its impact on women [3].

In 2016, World Health Organization (WHO) ranked Nepal the third highest in South Asia for female suicide mortality [4]. In Nepal, suicide is the single leading cause of death among women of reproductive age (WRA) [5]. Since suicide is considered an illegal act, requiring reporting to police, there is likely a gross underestimation of suicide cases reported by the police. Although, police data is the official source of suicide data in Nepal, some national mortality surveys have also been found collecting information on suicide. In absence of a national-level suicide surveillance system Nepal lacks reliable data on suicide [6]. Furthermore, the suicide-related indicators reported by Ministry of Health and Population are not reliable [6], demonstrating an obscure picture on the magnitude of suicide, and its burden among women in Nepal. In addition, the majority of the studies conducted focused on suicide in general. This reveals the significance of the problem of women’s suicide in Nepal and the need of a holistic approach to understand the depth of this issue.

Scoping review is recommended in settings with limited evidence on a subject as it covers a wider range of related topics [7]. A scoping review helps in mapping the key concepts underpinning a research area. The significance of it in evidence-based practice is to explore the extent of the literature on a specific topic along with research findings and gaps [7]. Considering the scattered evidence available on suicide among women in Nepal, we conducted a scoping review by reviewing both published (i.e. peer reviewed journals) and unpublished studies (i.e. reports not published in academic literature). The review was guided by a research query based on the notion what is known about suicide and deliberate self-harm among women in Nepal from the existing literature. The aim of this scoping review was to map the existing literature providing a more holistic view on suicide/deliberate self-harm (DSH) patterns, means of suicide and contributing factors of suicide among women in Nepal in order to understand the various contexts related to vulnerability of Nepalese woman towards suicide and deliberate self-harm. Further, the scoping review will act as a stepping stone for an ethnographic study in this area.

## Methods

To understand the context of suicide among women in Nepal, a scoping review of peer-reviewed publications and grey literature search from public domain on internet was conducted to map the existing literature on suicide/deliberate self-harm (DSH) patterns, means of suicide, and contributing factors to suicide among women in Nepal.

### Protocol design

Methods for this review, were developed following the six-step framework as per Arksey and O’Malley (2005) review methodology which includes: (i) identifying the research question; (ii) identifying relevant studies; (iii) study selection; (iv) charting the data; (v) collating, summarizing and reporting the results; and (vi) consultation. The optional “consultation exercise” of the framework was done by team consultation with a panel of two other authors AK and MvdP, who have expertise in this area, to validate the findings.

### Identifying the research question

The overall main research question developed to guide this review was: *What is known form the existing literature about suicide and deliberate-harm among women in Nepal?* Given the expansive nature of this type of review, outlined parameters were defined: *Women* represented females of all ages; *Suicide* referred to completed or attempted suicide and defined as an act of self-harm with or without fatal outcome; [8] and *Deliberate self-harm* was defined as a non-fatal act of self-harm carried out with variable motivations [8].

### Identifying relevant studies

Online National Library of Medicine’s PubMed and Google scholar search engines in July 2019 were used to identify peer reviewed publications. A Boolean search strategy combined key terms and operators: “suicide” AND “women” AND “Nepal”; “deliberate self-harm” AND “women” AND “Nepal”; “suicide” AND “women” and “Nepal” OR “deliberate self-harm” AND “women” AND “Nepal”. Internet-posted grey literature was searched for reports by government, national and international organizations.

### Study selection

Study eligibility was determined beginning with the screening of the title followed by the abstract scan, and finally a full-text review. To be eligible the studies needed to involve the following inclusion and exclusion criteria.

Inclusion criteria.

- Studies and reports on completed/attempted suicide or deliberate self-harm among Nepalese women across all ages including patterns, means of suicide and contributing factors.
- Any study designs such as primary research, case studies or reports.
- Papers published or reports issued between 2000 and 2019
- Full text articles

Exclusion criteria.

- Publications in languages other than English
- Book chapters, conference proceedings, editorials, dissertations and commentaries

By applying the eligibility criteria a single reviewer (SPK) screened the articles for selection. Two other authors (AK and MvdP) were consulted for calibration and validation. An inter-coder reliability check was done (by SPK and MvdP) using Holsti’s coefficient, testing 10% of the papers included in the analysis. This resulted in a Holsti’s coefficient of 0.9, where a coefficient of greater than 0.9 or greater is considered to represent high level of acceptability [9].

### Charting the data

Data retrieved from all articles selected for the final analysis, in line with the Arskey and O’Malley’s framework (2005), were extracted and entered in a data charting format, generated on Microsoft Excel spreadsheet, using the following categories: author, year of publication, study location, study design, study setting, sample size along with specific information on proportion of female participants and percentage of suicide cases. The spreadsheet was checked for comprehensibility and required modifications were implemented accordingly.

### Collating, summarizing and reporting the results

The findings from the collated data retrieved were then summarized into the following research domains: age, marital status, means of suicide, psychosocial and economic factors, mental health conditions and other factors.

## Results

**Fig. 1:**
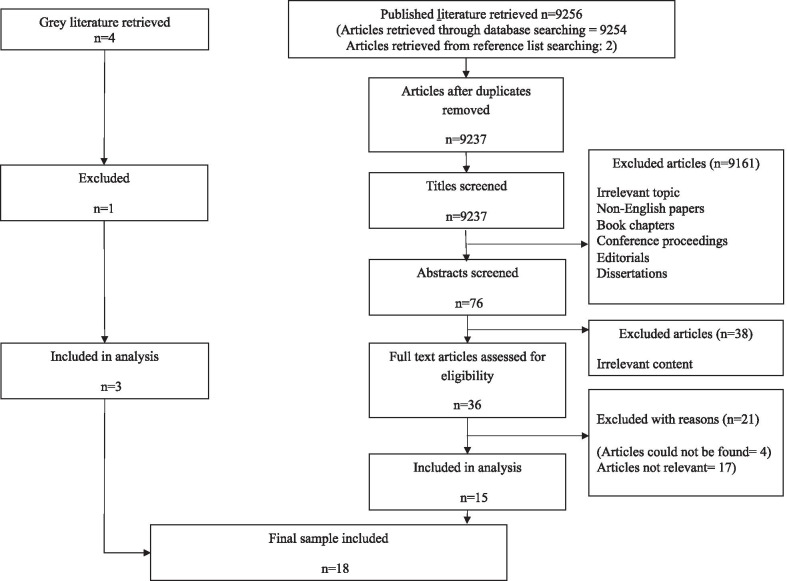
Review flowchart of searched literature

Electronic searches identified 9256 peer reviewed articles. Of these, 9233 peer review articles were retrieved from Google Scholar, 21 from online National Library of Medicine’s PubMed, and two articles from searching the reference list. Out of them, 9219 were omitted following exclusion of duplicated articles and screening of titles and abstracts for compliance with inclusion and exclusion criteria. The remaining 36 peer reviewed articles were read entirely, leaving 15 peer-reviewed articles which met all the inclusion criteria. Sample size, methodology and quality of the studies were not taken into account where possible. Three additional reports were identified through grey literature. Finally, 18 studies were selected (15 peer reviewed articles and 3 reports) for this review study (Fig. 1).

Fifteen peer reviewed studies were relevant to suicide among women in Nepal (see Table 1). The additional grey literature comprised of three reports (Table 2) conducted in community settings, using cross sectional, descriptive study, verbal autopsy and key informant interviews as study methods.

**Table 1:** Peer reviewed studies

S.N	Author/Year	Study location	Study design	Study setting	Sample size	Proportion of female participants		
						Male to Female ratio	Percentage	Number
1	Singh and Aacharya (2007)	Kathmandu	Retrospective case study	Hospital setting	99	1 to 1.3	–	–
2	Bajracharya et al. (2008)	Kathmandu	Retrospective case study	Hospital setting	78	–	–	57 females
3	Subba et al. (2009)	Western Nepal	Retrospective case study	Hospital setting	173	–	–	107 females
4	Sapkota et al. (2011)	Eastern Nepal	Cross sectional case study	Hospital setting	100	–	56% female	–
5	Agarwaal and Karki (2014)	Eastern Nepal	Retrospective case study	Hospital setting	1661	1 to 1.23	–	–
6	Ghimire et al. (2014)	Eastern Nepal	Cross sectional case study	Hospital setting	200	1 to 1.35	–	–
7	Pyakurel et al. (2014)	Far West Region Nepal	Cross sectional study	Community setting	11,737	–	100% female	–
8	Shakya (2014)	Eastern Nepal	Descriptive study	Hospital setting	115	0.74 to 1	–	–
9	Lama et al. (2015)	Kathmandu	Retrospective case study	Hospital setting	1148	N/A	N/A	N/A
10	Subedi, Chataut and Pradhan (2015)	Central Nepal	Retrospective case study	Hospital setting	470	1 to 1	–	–
11	Kafle, Bagale and Dhungana (2016)	Rupandehi	Cross sectional case study	Hospital setting	75	–	–	59 females
12	Gyenwali et al. (2017)	Chitwan	Cross sectional case study	Hospital setting	439	1 to 1.99	–	–
13	Hagaman et al. (2017)	Jumla and Kathmandu	Mixed method study	Community setting	302	1 to 1.32	–	–
14	Hagaman et al. (2018)	Jumla and Kathmandu	Mixed method study	Community setting	39	–	–	18 females
15	Thapaliya et al. (2018)	Southern Nepal	Retrospective case study	Hospital setting	116	68.1% female	–	–

**Table 2:** Grey literature reports

S.N	Author/year	Study location	Study design	Study setting	Sample size	Proportion of female participants		
						Male to Female ratio	Percentage	Number
1	Pradhan et al. (2010)	Maternal Mortality and Morbidity study from eight districts	Verbal autopsy of all maternal mortality cases	Community setting	239 suicide/1496 deaths	–	100% female	–
2	Pradhan et al. (2011)	Nepal	Literature review, key informant interview, qualitative and quantitative analysis	Community setting	N/A	–	100% female	–
3	Karki et al. (2017)	Ilam district	Cross–sectional, descriptive study	Community setting	1440	–	58.1% female	–

### Age

In a majority of the studies, more than 75% of the suicide/DSH attempts occurred below the age of 35 years [10–14] and around 60% under the age of 30 years [15–18]. Suicide data collected by the police in 2009/2010 demonstrated that the suicide rate peaked among women aged between 20–24 years at 7.4 per 100,000 [19]. The 2008/2009 Maternal Mortality and Morbidity Study (MMMS) showed that suicide was the leading cause of death in around 16% of the 1496 deaths of WRA (15–49 years). This was 6% more than the 10% of deaths in WRA in the 1998/1999 MMMS study. In the 2008/2009 MMMS the proportion of death due to suicide among younger women (15–34 years) was 24% compared to 8% for older women (35–49 years). In total, 63% of suicide deaths occurred among women 15–29 years old [5]. Furthermore, a 10-year study of 1148 burn cases admitted in a tertiary government hospital in Kathmandu, reported that 21% were female DSH burn victims between the age 20–30 years [20]. Additionally, a study conducted on the pattern and trend of deliberate self-harm in Western Nepal, where deliberate self-harm included parasuicide and suicide, disclosed that 82.3% of the DSH cases occurred among the 15–34 year age group with around 60% of them being female [16]. These results suggest a higher risk of suicide and DSH for young women between 15 and 35 years of age.

### Marital status

In most studies, the majority of suicide/DSH victims were married [11, 13, 14, 20, 21]. For example, in the 2008/2009 MMMS study, 73% of the women who committed suicide out of a total 239 suicide deaths among WRA were married in comparison to 20.9% of women who committed suicide were unmarried, and police reported for 2003–2011, 84.1% of all ages of women who committed suicide were married [5, 19]. Additionally, in the 2008/2009 MMMS study out of a total of 197 deaths to unmarried WRA, 50 deaths were due to suicide accounting for 25% of suicide deaths. Whereas, out of a total of 1191 deaths to married WRA, 174 deaths were due to suicide accounting for 15% of suicide deaths. This points out a higher proportion of suicide deaths among the unmarried WRA in comparison to the married WRA suggesting an increasing risk of suicide among unmarried WRA [5]. Therefore, although married women are at higher risk of suicide, suicide among unmarried WRA might be on the rise too. Furthermore, a study conducted on DSH at a tertiary referral center in Eastern Nepal reported that out of 200 cases 57.5% were female and 73.5% were married [11]. Similarly, in an another study conducted at B. P. Koirala Institute of Health Science (BPKIHS) in Dharan, Nepal, on suicidal attempts or DSH, out of 115 cases 57.39% were female and 69.6% were married.^[21]^Thus, these data indicate higher risk of suicide and DSH among married women in Nepal.

### Means of suicide

Poisoning stood out as the most common method of suicide and DSH in 88% of studies with most of the suicide victims consuming pesticide (commonly organophosphates) [11, 13–16, 22, 23]. In 16% of the studies, hanging was the method employed for suicide [12, 18, 24]. These findings were consistent with findings from the MMMS 2008/09. However, while 2008/2009 MMMS disclosed poisoning as the most common means of suicide, police data showed hanging as the most common means of suicide followed by poisoning [5, 19]. This may be explained by the fact that hanging cases have higher fatality and are more likely to be reported to the police. Whereas poisoning cases if referred to a hospital in time have a higher chance of survival and therefore may not be reported to the police [14, 19]. In the MMMS 2008/2009 drowning and burning were minor forms of methods accounting for 2% each [5]. According to the police data collected between 2009–2011, burning was three times more common as method of suicide among women than men [19] and a study conducted in a tertiary hospital in Kathmandu reported that among all burn patients, 79% were DSH or self-inflicted burns in females [20]. Furthermore, in studies conducted on DSH at a tertiary referral center in Eastern Nepal, Bir Hospital in Kathmandu, and four major hospitals (Bharatpur Hospital, Ratnanagar Hospital, Chitwan Medical College Teaching Hospital, College of Medical Sciences Teaching Hospital) in Chitwan, poisoning was the most common mode of DSH among women [11, 15, 17].

### Contributing factors

#### Psychosocial and economic factors

Abuse, interpersonal conflicts, marital disputes, relationship problems, and adjustment problems were the most common psychosocial factors reported, leading to suicide/DSH among Nepalese women [10, 11, 13, 18, 20, 21, 24, 25]. In a recent psychological autopsy study [18] based on suicide police cases between 2013/2015, 61.1% of the female suicide deceased were found to have undergone physical abuse within three months prior to suicide. For these women, abusers were husbands or partners, in-laws or paternal family (i.e. parents/siblings/relatives), or from the general community. However, husbands were the most common perpetrators of physical abuse [19, 25] and found to be involved in more than half of suicide cases due to family, marriage or relationship issues [5]. Domestic abuse usually affected married women and mostly co-occurred with husband’s excessive alcohol intake [25].

Interpersonal conflicts and marital dispute with husband and in-laws have been found to be most common stressors for DSH among women [11, 21]. Interpersonal conflicts with in-laws and marital disputes between spouses were typically related to accusations of husband/wife’s love affair; husbands alleged second marriage or extramarital affairs; husbands excessive alcohol consumption leading to increased stress and violence; and marrying at young age among the married women [10, 11, 13, 14, 20, 21, 24, 25]. A psychological autopsy investigation of suicide deaths in Nepal revealed that a woman’s move from her maternal household to her husband’s household often preceded suicide [25], denoting adjustment issues, family conflicts or marital disputes and abuse as possible underlying conditions.

Financial problems, such as unpaid dowry or loans, and financial loss due to husband’s alcohol abuse were found to be economic precursors of suicide/DSH among women [13, 25]. In a psychological autopsy investigation of suicide deaths, unpaid dowry or loans were found to lead to arguments, fights and abuse between husband and wife which resulted in women attempting suicide when it became unbearable [25].

Among unmarried adolescent girls, interpersonal conflicts with parents and relationship issues with romantic partners were mostly related to forbidden love relationships, failure in romantic relationships, perceived rejection or verbal arguments with romantic partners, and academic failures [10, 11, 13, 24, 25].

### Mental health conditions

Mental health disorders were found to be one of the factors related to suicide/DSH among women in around one third of the studies [10, 11, 13, 18, 21]. Mood disorder (mainly depression) constituted a common mental health disorder related to suicide [13, 18, 21]. In the 2008/2009 MMMS, depression was the only disorder reported related to suicide among WRA [4]. Adjustment disorder and substance abuse disorders were conditions revealed in other studies to be relevant to suicide among women [10, 11, 13, 24].

### Other factors

Out of the 18 studies, two pointed out poverty, [18, 19] and only one study explored social exclusion, inequality, gender and women’s status, and lack of education as precursors of suicide among women in Nepal [19].

## Discussion

The results from this review provide an overview of the information about suicide and DSH among women in Nepal based on available literature. Findings from this study suggest that suicide and DSH among women is a worrisome public health problem in Nepal due to various underlying socio-cultural, economic and environmental factors. Based on our review, although suicide can occur at any age during the lifespan of an individual [1], suicide and DSH attempts are higher among younger females aged between 15–35 years compared to older ones [11, 15–18]. According to the MMMS (2008–2009), suicide stood out as the single leading cause of death among WRA in Nepal with 63% of suicide deaths occurring among women between 15–29 years [5]. Various studies conducted on DSH also highlighted higher DSH among married women aged between 15–30 years [11, 15, 17, 20, 21]. This apparently highlights the severity of suicide and DSH as a major health challenge with young women observed to be more vulnerable to suicide in comparison to older women.

These findings are similar to suicide among women in India where young women below the age of 30 are at higher risk of committing suicide [26]. Suicide among women in Asia may well be related to lack of the awareness of women’s rights, women’s dependency on men, and women’s social status. As women in Asian countries often have a subordinate position, they may experience high levels of stress which are intensified by family hierarchy and dynamics in societies [26]. Younger individuals are not sufficiently mature to handle stressors in general, and young women in Asia, including Nepal, face higher social, emotional or financial dependency on their families and husbands, which makes them vulnerable to such stressors with no one to turn to, potentially leading them towards DSH or suicide as a cry for help or a perceived route of escape. The reviewed literature identified young women to be more vulnerable to suicide compared to older women indicating age as a risk factor. Nonetheless, the studies do not explore in depth the conditions and reason behind it, untangling whether it is the younger age or rather the conditions and circumstances in the given age which could be the actual risk factor.

In most of our reviewed studies the majority of the suicide and DSH victims were found to be married (i.e., up to 84% in a review of police records) [5, 19]. In Nepal, as in other South Asian Countries, it is common for women to get married at a young age through arranged marriages with often a large age gap between husband and wife. As divorce is culturally demeaning and highly stigmatized, Nepalese women will stay married even after enduring abuse in an unhappy marriage [19]. Young women’s household decision-making capability and the challenges they face in dealing with the distribution of power and the dynamics within the in-law family are shaped by socio-cultural factors. This in turn not only increases their vulnerability towards being overburdened with domestic responsibilities and psychological abuse, but also physical violence [19]. Along with this, married South Asian women may also endure other pressures such as young motherhood, low social status and economic dependency making them susceptible to suicidal ideation and acts [27]. Nepalese women’s lower status in the family and society are reinforced by religious, cultural and social norms which could act as triggers of violence [28]. Therefore, in most South Asian countries, including Nepal, marital status does not appear to be a protective factor but rather a condition that seems to expose them to abuse, resulting in increased risk of suicide. The reviewed studies highlight being married as an underlying reason for suicide among women. However, the studies do not provide any clarity on whether it is the marital status itself or the stressful encounters endured in one’s married life which makes them vulnerable to suicide.

Although married women are at higher risk of suicide in Nepal, suicide among unmarried women is also on the rise. According to the MMMS 2008/09 even though suicide deaths were mostly persistent among married WRA, reflecting the larger number of married women in the society, suicide deaths accounted for a larger proportion in unmarried (25%) WRA than the married women (15%) WRA [5]. Traditionally, Nepal’s patriarchal and conservative perspective regarding marriage restricts youths’ involvement in their own life partner selection [29]. However, in recent times, family pressure on young unmarried girls for marrying the men chosen by parent’s agreement without considering their preference, results in a sense of loss of control over their own lives that can trigger suicidal attempts as the only option to get heard [19]. Other contributing reasons might be failed love relationships, lack of marriage building stress in the family, and pregnancy before marriage [19].

In our review, poisoning was the most common method for suicide and DSH among women with most of the suicide/ DSH victims consuming pesticide (commonly organophosphates) [11, 13, 15, 16], followed by hanging [12, 18, 24]. As women may attempt suicide/DSH as a means of expressing their need for attention and assistance [19], it could explain the use of less lethal methods for suicide, which is similar to observations across the globe. It seems easy access to pesticides and ropes and scarves [15, 16, 21] facilitates these methods of suicide.

Poisoning also offers the possibility of dosing intake, making it a preferred method when the intent is to alert the family of one’s distress without terminating one’s life. Furthermore, lack of understanding about the lethality of a given method may come into play as well [19]. This coincides with studies conducted in Sri Lanka that reported on women attempting suicide by ingesting pesticides or setting themselves on fire, with the intent to threaten the family members, but unaware of the risks involved leading towards lifelong irreversible impairment [30].

In the majority of studies reviewed, abuse, interpersonal conflicts, marital disputes, relationship problems and adjustment problems were the most common psychosocial factors leading to suicide/DSH among Nepalese women [10, 11, 13, 18, 20, 21, 24, 25]. While in-laws were found to be mostly responsible for emotional or physical abuse, the most common perpetrators of physical abuse were husbands [19, 25]. Findings from Nepal Demographic and Health Survey 2016 indicated among WRA, 22% of women and 26% of ever married women had experienced physical and spousal violence respectively at least once in their lifetime [31]. In Nepal, 60% of women have not sought help or disclosed to anyone the domestic violence they experienced in their lifetime [31]. Many Asian societies culturally disapprove discussion of domestic problems outside of the family, thus discouraging women from reporting abuse due to fear of public humiliation, shame and further retribution from their spouse [32]. As a result, not seeking any sort of assistance from any source leads to internalizing stresses and increasing the risk of suicide ideation, DSH and suicidal attempts.

Among married women interpersonal conflicts with in-laws and marital disputes between spouses were generally related to being married at a very young age, accusations of love affairs, husbands alleged second marriage or extramarital affairs and alcohol abuse leading to increased stress and violence [13, 14, 19, 25]. Nepalese women often indicate a husband’s alcohol abuse as an attribute to spousal violence and marital disputes [25, 33]. Whereas, among the unmarried adolescent women, interpersonal conflicts with parents were mostly over relationship problems and romantic partners, and academic failures [10, 11, 13, 14, 18, 20, 21, 25]. Lack of liberal perception regarding love relationships and inter-caste marriage, and non-acceptance of such relationships may lead to interpersonal disputes among young women and their families and/or their romantic partners. Academic failure is also a trigger for suicide and suicidal attempts among adolescent women [19]. A study on poisoning cases in Dhulikhel hospital, where the majority of cases were female, 16.67% consumed poison due to failure in examinations [34]. Further, the police records found the rate of suicide among youth to be higher during the School Leaving Exam (SLC/ Standard 10^th^ grade exam) and after the publication of its results [19].

Financial issues such as unpaid dowry and loans were found to be the economic factors resulting in suicide and DSH among women [13, 25]. Although women devote a lot to household wellbeing and income, they are not granted economic value or reward making them financially dependent and inferior to their husbands [19, 31]. Where women are financially dependent, it can be assumed that financial constraints due to husband’s alcohol abuse, and unpaid debts pose economic stressors. Such economic stressors usually take form of arguments, conflicts and abuse between spouses [25] and may result in suicide attempts or DSH among women.

Suicide is more closely connected to impulsivity than deliberate attempts or mental illness in Nepalese society [25]. A sense of hopelessness and despair relevant to domestic violence and social shame based on moral judgment from society appears to leave women to believe they have no other option than suicide as the only way out [25]. These findings are consistent with studies in India, where women consider suicide as their only option [35, 36]. This illustrated how emotional and social stress is internalized to act as a psychological stressor for impulsive suicidal attempts and DSH among women.

Mental health disorders, commonly mood disorders (such as depression) and adjustment disorder along with substance abuse were found to be relevant to suicide/DSH among women [10, 11, 13, 18, 21]. Mental illness could be interlinked with socio-economic factors of suicide, since poor mental health is an outcome of social determinants like poverty, social exclusion, marital problems, unemployment, low socio-economic status and stressful events [37, 38]. The 2008/09 MMMS reported that mental health problems were a major underlying factor due to repercussion of social determinants and demonstrated how social norms, poverty and poor access to healthcare overlap with poor mental health. Some cases in the study also highlighted how issues related to marriage (e.g., early marriage, or being deprived of love marriage) lead to depression [19].

Psychosocial and economic factors such as interpersonal conflicts, marital disputes, abuse, relationship problems, adjustment issues and financial problems; and mental health conditions were highlighted as factors associated with suicide and DSH among the majority of the studies reviewed. These present proximal factors related to suicide among women. Only a few studies were found to superficially explore factors such as poverty, social exclusion, gender inequity, and education which may be the root causes of suicide and subtly touched upon traditional, cultural and patriarchal systems in Nepal. For example Pradhan et al. pointed out that problems related to marital, husband/family relationship are related to women’s status and gender based violence which is influenced by the Nepalese socio-cultural gender norms and gender inequality [19].

The question arises as to why root causes of suicide receive limited attention. Is it due to the complexity of the issues involved and challenges presented when attempting to investigate these factors? Further, most of the studies reviewed took place in hospital settings, demonstrating the need for suicide studies focusing on community settings. Grassroot studies should embody a dialectical interface between women, partners and family and their socio-cultural environment to enhance insight in the dynamics of partner and family relationships and disentangle pathways in coping with stressors associated with suicide among young women in Nepal, informing future community-level suicide prevention efforts.

Addressing the emerging problem of suicide among women, calls for multidimensional approaches aimed at changing the socio-cultural environment and interpersonal relationships. Such approaches require policy responses and collaborations among government, civil society and communities. However, societal changes take time and resolution may unfold over a generation. While acknowledging the need for multidimensional approaches to suicide prevention, it became clear from our review that sociocultural and economic contexts shape family and marital relationships, impact personal mental wellbeing of Nepalese women and fuel suicidal attempts and DSH. Hence, grassroots study approaches involving husbands or partners and families could facilitate a deeper understanding of factors associated with suicide among women and be instrumental in exploring pathways for suicide prevention at community level.

There are some limitations in study design and analysis that could affect our findings: (i) only English publications were included, and information reviewed was limited to sources accessible via search engines and public domains; (ii) the study did not assess religious, ethnic and educational aspects as potential factors associated with suicide and DSH among women in Nepal due to paucity of information.

The study also possesses an important methodological strength: data analysis comes from reliable, and peer- reviewed journal publications and documents thus limiting the chances of using unreliable data or information in the analysis.

## Conclusion

Suicide and DSH among young women is a worrisome public health problem in Nepal. In absence of a national-level suicide surveillance system Nepal lacks reliable data on suicide, and the suicide-related indicators reported by Ministry of Health and Population are unreliable [6] leading to an unclear picture of the magnitude of suicide and its burden among women in Nepal.

Although married women are at higher risk of suicide and DSH in Nepal, unmarried women also appear to become increasingly vulnerable as pointed out by the available data. Socio-cultural and economic factors propel psycho-social and mental wellbeing of women in Nepal which is then associated with suicide and DSH. In a societal context characterized by patriarchy, gender inequity, poverty and rigid socio-cultural norms; family and marital relationships often result in adjustment problems, abuse, interpersonal conflicts, relationship problems, marital disputes and financial challenges. These stressors can have an adverse impact on mental wellbeing of young women in Nepal, turning them vulnerable to suicidal ideation, suicidal attempts and DSH.

Studies reviewed were limited to describing associations between proximal factors and suicide and DSH among Nepali women. Very few studies touched upon underlying social determinants, while no studies were found focusing on the dialectical interface between family members and their socio-cultural contexts and how this affects vulnerability of young women towards suicidal and DSH risks. A community-based study approach involving young women, husbands/partners and families could contribute towards a deeper understanding of factors associated with suicide, their dynamics and how they affect young women’s personal lives in both individual and community level. Additionally, exploring how socio-cultural and environmental context relate to the root causes such as patriarchy, gender inequity, poverty; and proximal causes among young women in Nepal, using a qualitative study approach could provide in-depth understanding of the situation. Qualitative studies including ethnographic or narrative research approaches could be useful in exploring these contexts. Such study approaches could provide insight on the influence of the socio-cultural environment on the perceptions of young women, husbands or partners, families and the community towards these stressors; help seeking behavior and the coping mechanisms adopted by women. This could lead to exploring potential pathways to mitigate the impact the socio-cultural environment and the stressors it produces for young married women in Nepal to inform community-based suicide prevention strategies.

## Data Availability

The datasets used and analyzed in the study are available from the corresponding author on reasonable request.

## Abbreviations

WHO: World health organization
WRA: Women of reproductive age
DSH: Deliberate self-harm
MMMS: Maternal mortality and morbidity study
